# Plasma proteomic profiling in dogs with pheochromocytoma

**DOI:** 10.1093/jvimsj/aalag212

**Published:** 2026-09-21

**Authors:** Marit F van den Berg, Alexandro Rodríguez-Rojas, Pavlos G Doulidis, Luciano Pisoni, Iwan A Burgener, Sara Galac

**Affiliations:** Department Clinical Sciences, Faculty of Veterinary Medicine, Utrecht University, 3584 CM Utrecht, The Netherlands; Division for Small Animal Internal Medicine, Department for Companion Animals and Horses, University of Veterinary Medicine Vienna, 1210 Vienna, Austria; Division for Small Animal Internal Medicine, Department for Companion Animals and Horses, University of Veterinary Medicine Vienna, 1210 Vienna, Austria; Department of Veterinary Medical Sciences, Faculty of Veterinary Medicine, University of Bologna, 40064 Ozzano dell’Emilia, Italy; Division for Small Animal Internal Medicine, Department for Companion Animals and Horses, University of Veterinary Medicine Vienna, 1210 Vienna, Austria; Department Clinical Sciences, Faculty of Veterinary Medicine, Utrecht University, 3584 CM Utrecht, The Netherlands

**Keywords:** adrenal, biomarker, dog, protein, proteome, therapeutic target

## Abstract

**Background:**

Pheochromocytomas (PCCs) in dogs are challenging to diagnose. Plasma proteomics offers a minimally invasive approach to identify circulating biomarkers and disease-relevant pathways.

**Hypothesis/Objectives:**

To compare the plasma proteome of dogs with PCC and controls to (1) identify differentially abundant proteins, (2) characterize altered pathways, and (3) nominate candidate circulating biomarkers and therapeutic targets.

**Animals:**

Plasma from 10 client-owned PCC dogs and 10 healthy controls.

**Methods:**

Multicenter, retrospective, observational, exploratory study using label-free liquid chromatography-mass spectrometry. Primary outcomes were the identification of differentially abundant proteins and characterization of altered pathways and protein functions.

**Results:**

Principal component analysis demonstrated clear separation between PCC and control dogs. Of 261 reliably quantified proteins, 51 were differentially abundant (false discovery rate-adjusted *P* < .05). Of these, 33 had a log_2_ fold change of >1 or < −1, with 15 showing higher and 18 lower plasma abundance in PCC. Compared with controls, PCC dogs showed increased abundance of proteins linked to cell adhesion/migration and metastatic potential, hemostasis, and regulation of apoptotic signaling, alongside alterations in oxidative stress and metabolic processes. Several proteins with higher abundance, including CD44, peroxiredoxin-2, and peptidyl-prolyl cis-trans isomerase, emerged as promising candidates for therapeutic exploration.

**Conclusions and clinical importance:**

Dogs with PCC exhibit a distinct circulating protein signature vs healthy controls, providing clues to PCC pathogenesis and nominating proteins as diagnostic biomarker candidates and potential therapeutic targets.

## Introduction

Pheochromocytomas (PCCs) are neuroendocrine tumors arising from chromaffin cells of the adrenal medulla that typically secrete excess catecholamines. Affected dogs can present with mild and non-specific clinical signs, such as lethargy, polyuria/polydipsia, vomiting, hyporexia, or abdominal pain, as well as potentially life-threatening cardiovascular manifestations including collapse, systemic hypertension, and arrhythmias. Additional problems can result from mass effect or locally invasive growth.^1–3^

Clinical diagnosis of PCC can be challenging because presenting signs overlap with cardiovascular, neurologic, or endocrine disorders, catecholamine secretion can be intermittent, and a subset of tumors are “biochemically negative”, with plasma or urinary metanephrines within reference intervals despite the presence of PCC.^4^^,^^5^ Identification of an adrenal mass requires abdominal ultrasonography by an experienced operator or access to advanced imaging such as computed tomography. Measurement of plasma or urinary metanephrines is the recommended biochemical test; however, overlap with non-PCC conditions and the occurrence of biochemically negative tumors limit its stand-alone utility. Consequently, definitive diagnosis still relies on histopathology and immunohistochemistry (IHC).^5^ Therapeutic options are limited. Adrenalectomy is the treatment of choice but is not feasible for all dogs due to extensive local tumor invasion, distant metastasis, or severe concurrent disease. Beyond limited experience with tyrosine kinase inhibitors in small cohorts, no established drug therapies exist.^6^^,^^7^ Accordingly, there is a need to deepen biological understanding of PCC to enable new, targeted treatments, alongside improved diagnostic tools for earlier and more accurate detection.

Proteomic analysis offers such an avenue. Untargeted, mass-spectrometry–based plasma proteomics can quantify hundreds of proteins in parallel with peptide-level specificity, enabling unbiased discovery of candidate biomarkers, mapping of affected pathways, and nomination of therapeutic targets; as a blood-based, minimally invasive approach, it is well suited to clinical translation.^8^ In dogs, proteomic profiling has been reported across several diseases,^9–11^ but not for PCC. Therefore, the aim of this study was to compare the plasma proteome of dogs with PCC and healthy controls, delineate altered biological pathways, and nominate candidate circulating biomarkers and potential therapeutic targets for future validation.

## Materials and methods

### Study design, study population, and sample collection

This multicenter, retrospective observational study compared plasma proteomic profiles between dogs with PCC and healthy controls. Surplus plasma from ten dogs with histologically confirmed PCC and ten clinically healthy dogs was included. Medical records were retrospectively reviewed. Controls were deemed healthy based on history, physical examination, and blood analysis (complete blood count (CBC) and biochemistry). Exclusion criteria included age <1 year, body weight <3 kg, any clinical abnormality, medication use within the last 2 months, or abnormal laboratory values. Written informed consent was obtained from all owners. Plasma was stored at −21 °C until analysis. Additional information is provided in the Appendix.

### Primary outcomes

Primary outcomes were the identification of differentially abundant plasma proteins between PCC dogs and controls, and the characterization of altered biological pathways and functional categories.

### Label-free liquid chromatography-mass spectrometry (LC–MS) plasma proteomics: sample preparation, data processing, and statistical analysis

Plasma proteins were extracted, digested to peptides, and analyzed by label-free quantification LC–MS. Detailed protocols, LC–MS acquisition parameters, and quality control procedures are provided in the Appendix. Raw MS data were analyzed using MaxQuant (version 2.6.6.0)^12^ with the integrated Andromeda search engine and searched against the *Canis lupus familiaris* reference proteome. Data filtering and statistical analysis were performed using Perseus (version 2.1.3.0).^13^ Differentially abundant proteins were identified using Student’s *t-*test with false discovery rate (FDR) correction in Perseus. Proteins with a log₂ fold change (FC) >1 or < -1 and an FDR-adjusted *P* value ≤ .05 were considered significantly abundant. All statistical analyses were performed using R (version 4.4.2).

### Bioinformatics

Gene ontology (GO) classification (PANTHER)^14^ was performed for significantly differentially abundant proteins (FDR-adjusted *P* < .05, log₂FC > 1 or < -1) and summarized by biological process (BP), molecular function (MF), protein class, and pathway. Protein interaction networks were generated using the STRING database (version 12.0; https://string-db.org)^15^ for *C. lupus familiaris*. Functional enrichment analyses were conducted in the Proteomics Research Environment (ProteoRE).^16^^,^^17^ Significantly differentially abundant proteins were compared to a reference set of proteins comprising all identified and quantified proteins. Both Singular Enrichment Analysis (SEA) and Modular Enrichment Analysis (MEA) were applied for the GO domains BP, MF, and cellular component (CC). Direction-specific comparisons of proteins with increased vs decreased abundance were performed using the Biological theme comparison (clusterProfiler) tool in ProteoRE. Full details are provided in the Appendix.

## Results

### Animals

Ten dogs diagnosed with PCC were included, consisting of 4 females (all neutered) and 6 males (2 neutered, 4 intact). Median age at the time of blood sampling was 11.2 years (range: 9.6-13.0). Adrenalectomy was performed in 9 dogs; 1 dog (PCC2) with bilateral adrenomegaly and concurrent tracheobronchomalacia was treated medically with phenoxybenzamine. At the time of diagnosis and blood sampling, comorbidities were present in several dogs with PCC. PCC1 had chronic enteropathy, well controlled with a monoprotein home-prepared diet and probiotic supplementation, and a hormonally inactive thyroid nodule. PCC2 had tracheobronchomalacia. PCC3, PCC8, and PCC9 had no relevant comorbidities reported. PCC4 had a history of pneumothorax due to pulmonary bullae, without ongoing clinical signs at the time of sampling. PCC5 had a mammary carcinoma that had been completely resected before PCC diagnosis. PCC6 had syringomyelia and a cervical intervertebral disc herniation diagnosed several years earlier and managed with gabapentin, as well as myxomatous mitral valve disease (stage B2) diagnosed 1 year before PCC diagnosis and treated with pimobendan. PCC7 had chronic signs of gastrointestinal disease consistent with suspected chronic enteropathy, managed with a hypoallergenic diet and intermittent supportive therapy. Information on comorbidities was not available for PCC10. Clinical data, including age, plasma metanephrine concentrations, tumor characteristics, and presence of vascular invasion, are presented in Supplementary Table 1. Ten clinically healthy, client-owned dogs served as controls: 8 females (5 neutered, 3 intact) and 2 neutered males. Median age at sampling was 4.3 years (range: 1-10). Breeds represented included 4 mixed-breed dogs, 2 Poodles, and 1 each of German Shepherd Dog, Labrador Retriever, Siberian Husky, and French Bulldog. Control dogs were significantly younger than dogs with PCC (*P* < .001).

### Differentially abundant proteins

**Figure 1:**
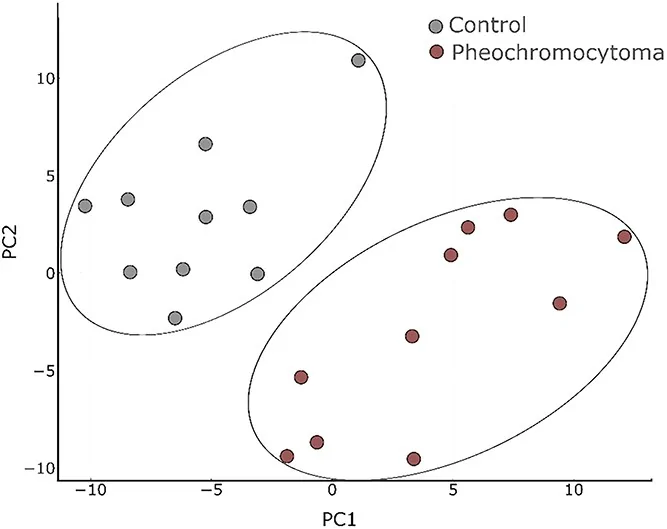
Principal component analysis shows that healthy control dogs clearly cluster apart from dogs with pheochromocytomas.

**Figure 2:**
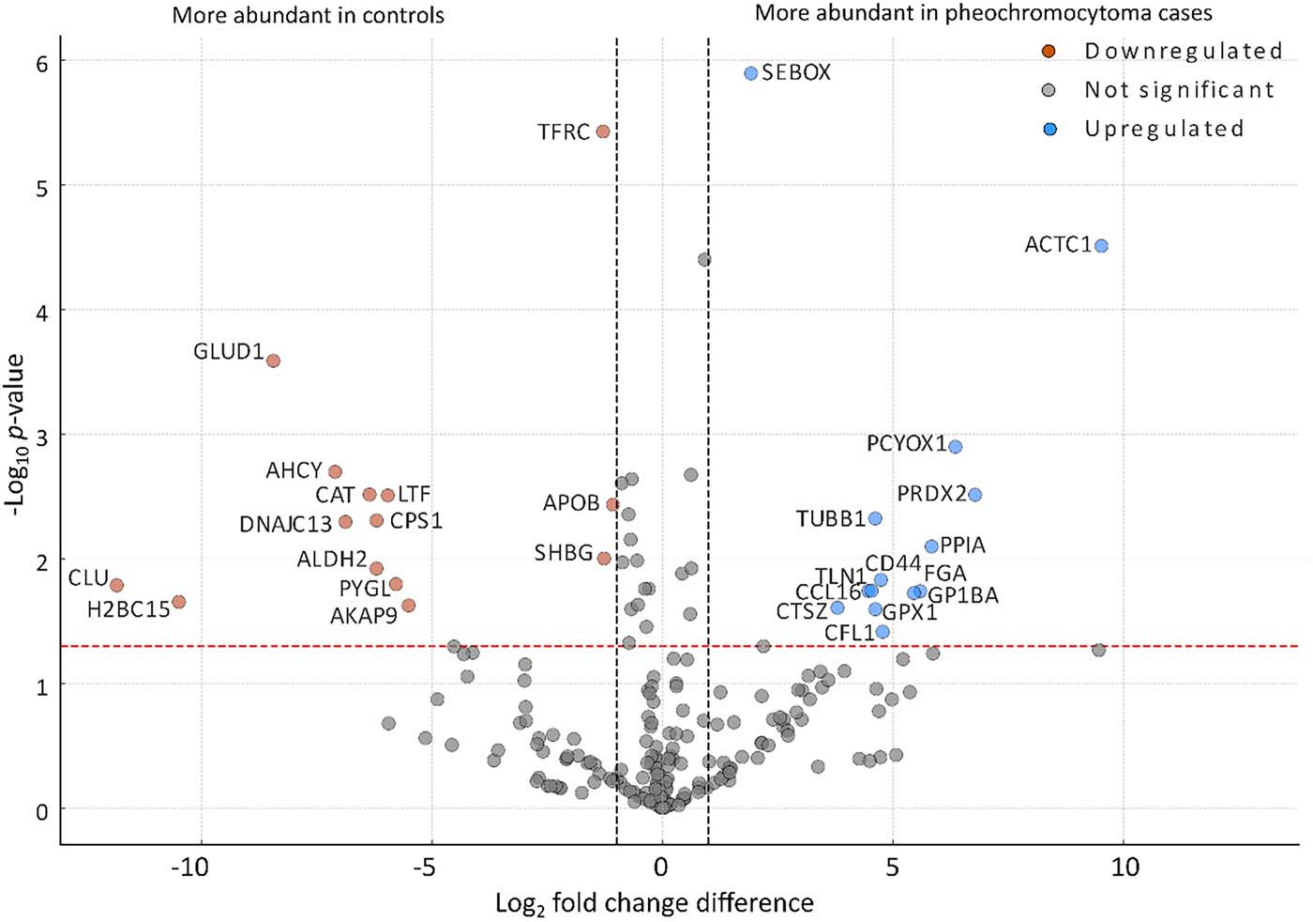
Volcano plot illustrating differences in plasma protein intensities between pheochromocytoma-affected dogs and healthy controls. Proteins with a minimum 2-fold change (log₂ FC ≥1 or ≤ −1) and an FDR-adjusted *P* ≤ .05 were considered significantly differentially abundant. Blue dots represent proteins with significantly higher abundance in dogs with PCC relative to controls, whereas red dots indicate proteins with significantly lower abundance. Gray dots below the red dotted line represent non-significant proteins (FDR-adjusted *P* > .05), while gray dots above the red dotted line represent proteins with a FDR-adjusted *P* ≤ .05, but log₂ FC between −1 and 1. Only identified proteins are shown; unidentified proteins were excluded from the plot.

Quantitative proteomic profiles were compared between dogs with PCC and healthy control dogs. Principal component analysis revealed a clear separation between the PCC and control groups, indicating distinct proteomic signatures (Figure 1). In total, 261 proteins were reliably quantified (Supplementary Table 2). Of these, 51 proteins had differential abundance between PCC-affected and control dogs based on an FDR-adjusted *P* < .05 (Supplementary Table 3). Among these, 33 proteins had a log₂ FC greater than 1 or less than −1. Specifically, 15 proteins were present at higher levels, and 18 were present at lower levels in plasma of dogs with PCC (Figure 2, Table 1). Heatmaps of the plasma proteins with increased and decreased abundance in dogs with PCC compared to healthy controls are shown in Supplementary Figures 1 and 2.

**Table 1:** Differentially abundant proteins in plasma of dogs with PCC in comparison with healthy controls. Listed are proteins with an FDR-adjusted *P* < .05 and a log₂ fold change > 1 (increased abundance) or < −1 (decreased abundance).

**Identified protein**	**Protein ID**	**Gene name**	**Log** _**2**_ **FC**	**FDR-adj.** ***P* value**
Uncharacterized Ig-like domain-containing protein^*^	A0A8I3P6S7 (+3)		9.60291	.014642
**Actin, alpha cardiac muscle 1**	A0A8I3QWF2 (+6)	ACTC1	9.52338	.000157
**Peroxiredoxin-2**	A0A8I3NZ15 (+1)	PRDX2	6.78120	.006154
**Prenylcysteine oxidase 1**	A0A8I3Q395	PCYOX1	6.35400	.003007
**Peptidyl-prolyl cis-trans isomerase**	A0A8I3NVG7 (+7)	PPIA	5.83841	.009354
**Fibrinogen alpha chain**	P68213	FGA	5.59142	.018254
**Platelet glycoprotein Ib alpha chain**	Q28256	GP1BA	5.45371	.022950
**Cofilin-1**	A0A8I3P3F3 (+10)	CFL1	4.77705	.042098
**CD44 antigen**	Q28284 (+8)	CD44	4.73798	.024398
**Glutathione peroxidase**	A0A8I3PT87	GPX1	4.61704	.031118
**Tubulin beta chain**	A0A8I3NIF5	TUBB1	4.61506	.005607
**Talin 1**	A0A8I3N6K0 (+1)	TLN1	4.53658	.021101
**C-C motif chemokine**	A0A8I3N166	CCL16	4.46581	019219
**Cathepsin Z**	A0A8I3S0K4 (+2)	CTSZ	3.79417	.026845
**Vitronectin**	A0A8I3N025 (+2)	SEBOX	1.92140	.000002
				
**Apolipoprotein B**	A0A8I3S4D1 (+1)	APOB	−1.07621	.003706
**Sex hormone-binding globulin**	A0A8I3PLX4 (+4)	SHBG	−1.26907	.010859
**Transferrin receptor protein 1**	A0A0A0RBT6 (+1)	TFRC	−1.29222	.000008
**Uncharacterized Ig-like domain-containing protein^a^**	A0A8I3QLC2		−4.98108	.036917
**Uncharacterized IgG-like domain-containing protein^a^**	A0A8I3SCG6 (+1)		−5.43380	.025455
**A-kinase anchoring protein 9**	A0A8I3NM97 (+1)	AKAP9	−5.51112	.025031
**Glycogen Phosphorylase L**	A0A8I3RRB6 (+8)	PYGL	−5.78649	.016150
**Uncharacterized protein**	A0A5F4BYG2		−5.89170	.013792
**Lactotransferrin**	A0A8I3NMR3 (+3)	LTF	−5.96278	.003185
**Carbamoyl-phosphate synthase 1**	A0A8I3P9N3 (+1)	CPS1	−6.20513	.005814
**Aldehyde dehydrogenase 2 Family Member**	A0A8I3PA71 (+4)	ALDH2	−6.20685	.015885
**Catalase**	O97492	CAT	−6.35732	.004735
**Uncharacterized Ig-like domain-containing protein^a^**	A0A8I3P1K6 (+1)		−6.74507	.041297
**DnaJ heat shock protein family (Hsp40) member C13**	A0A8I3Q663 (+1)	DNAJC13	−6.88204	.010641
**Adenosylhomocysteinase**	A0A8I3NY33 (+1)	AHCY	−7.10559	.002013
**Glutamate dehydrogenase 1**	A0A8I3N1X6 (+2)	GLUD1	−8.44577	.000647
**Histone H2B**	J9P6S8 (+8)	H2BC15	−10.49660	.025629
**Clusterin**	P25473 (+4)	CLU	−11.84407	.018467

Abbreviations: FC = fold change; FDR = false discovery rate; PCC = pheochromocytoma.

a Multiple proteins annotated as “Ig-like domain-containing protein” correspond to distinct UniProt entries with limited functional annotation in the canine reference proteome.

Among the plasma proteins with increased abundance in dogs with PCC, several were closely associated with cell adhesion, migration, and metastatic potential, including cofilin-1, CD44, talin 1, peptidylprolyl isomerase A (cyclophilin A), and cathepsin Z. Proteins linked to hemostasis (fibrinogen α-chain, platelet glycoprotein Ib α) were also elevated in plasma. In addition, a subset of increased proteins was related to oxidative stress response and antioxidant activity. Notably, peroxiredoxin 2 and glutathione peroxidase 1, both key antioxidant enzymes, showed higher plasma concentrations in dogs with PCC. Among the proteins with reduced plasma abundance, several were involved in iron handling (transferrin receptor 1, lactotransferrin) and lipoprotein transport/homeostasis (apolipoprotein B, clusterin). In addition, multiple proteins were linked to antioxidant defense (eg, catalase and aldehyde dehydrogenase 2), and to glutamine-glutamate and amino acid metabolism (glutamate dehydrogenase 1, adenosylhomocysteinase, carbamoyl-phosphate synthase 1).

To gain a broader functional overview of the differentially abundant proteins, GO classification and enrichment analyses were performed.

### Gene ontology classification

**Figure 3:**
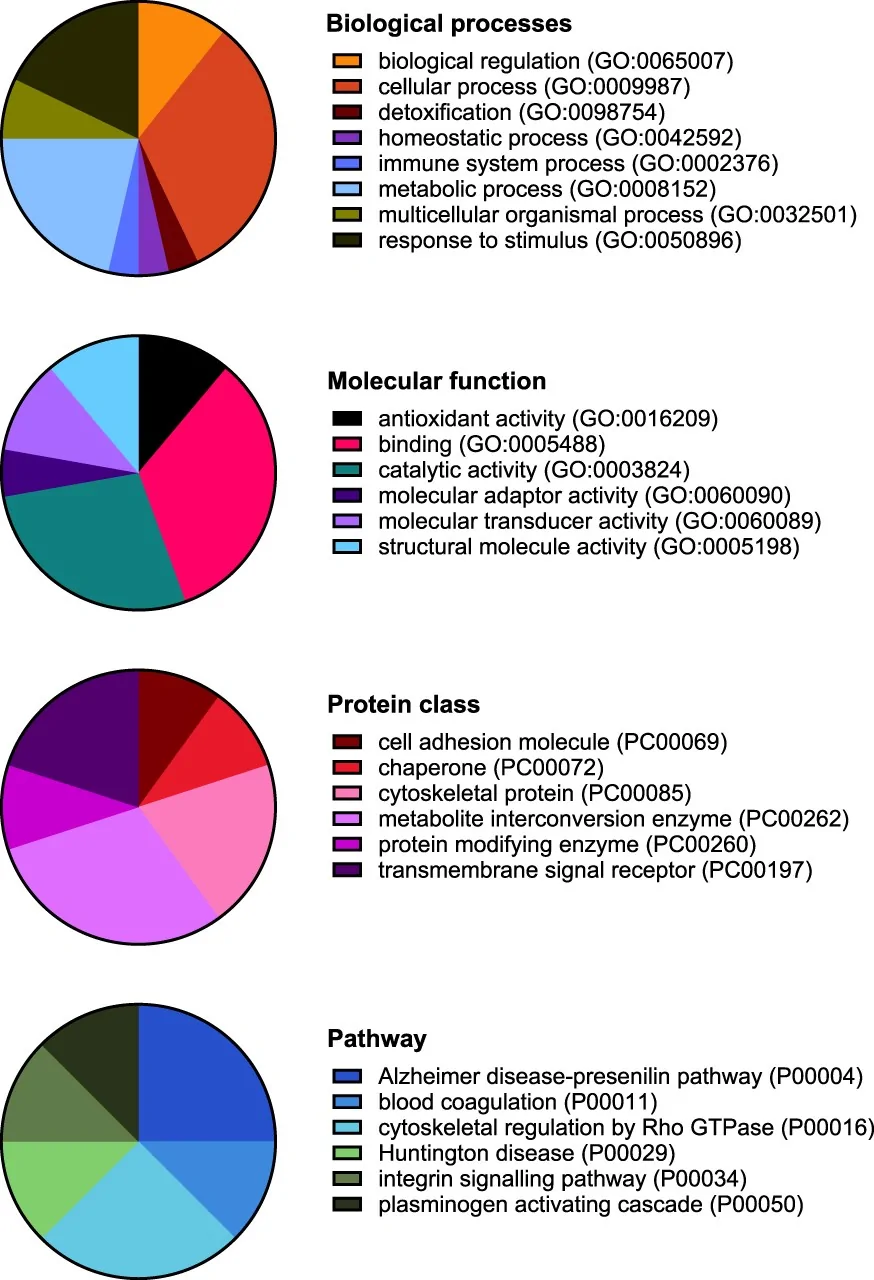
Gene ontology analysis of proteins elevated in dogs with pheochromocytomas in comparison with healthy controls. Proteins with significantly increased abundance (false discovery rate-adjusted *P* < .05, log₂ FC >1) were classified using PANTHER according to biological process, molecular function, protein class, and pathway.

**Figure 4:**
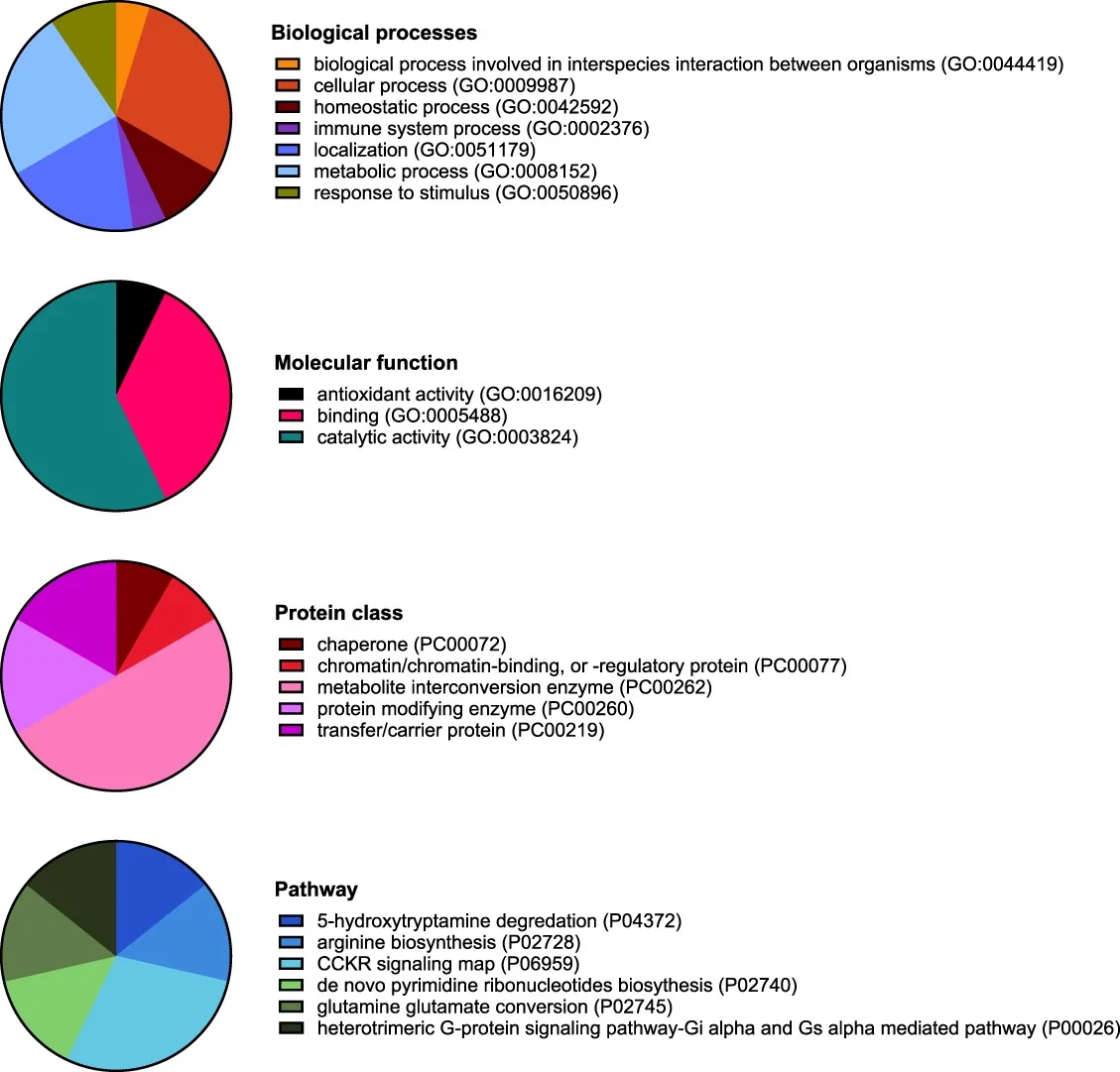
Gene ontology analysis of proteins reduced in dogs with pheochromocytomas relative to healthy controls. Proteins with significantly decreased abundance (false discovery rate-adjusted *P* < .05, log₂ FC < −1) were classified using PANTHER according to biological process, molecular function, protein class, and pathway.

Gene ontology classification revealed that proteins with increased abundance in dogs with PCC were mainly involved in cellular and metabolic processes, and were enriched for catalytic and binding activities (Figure 3). Pathway categorization indicated predominant involvement of cytoskeletal regulation by Rho GTPase, integrin signaling, plasminogen activation, and blood coagulation pathways. Proteins with decreased plasma abundance in dogs with PCC were primarily associated with cellular and metabolic processes, as well as localization (Figure 4). At the molecular level, these proteins mainly exhibited catalytic and binding activities. Pathway categorization indicated reduced activity in CCKR signaling and in amino acid and nucleotide metabolism, including arginine biosynthesis and pyrimidine ribonucleotide biosynthesis.

### Protein–protein interaction

**Figure 5:**
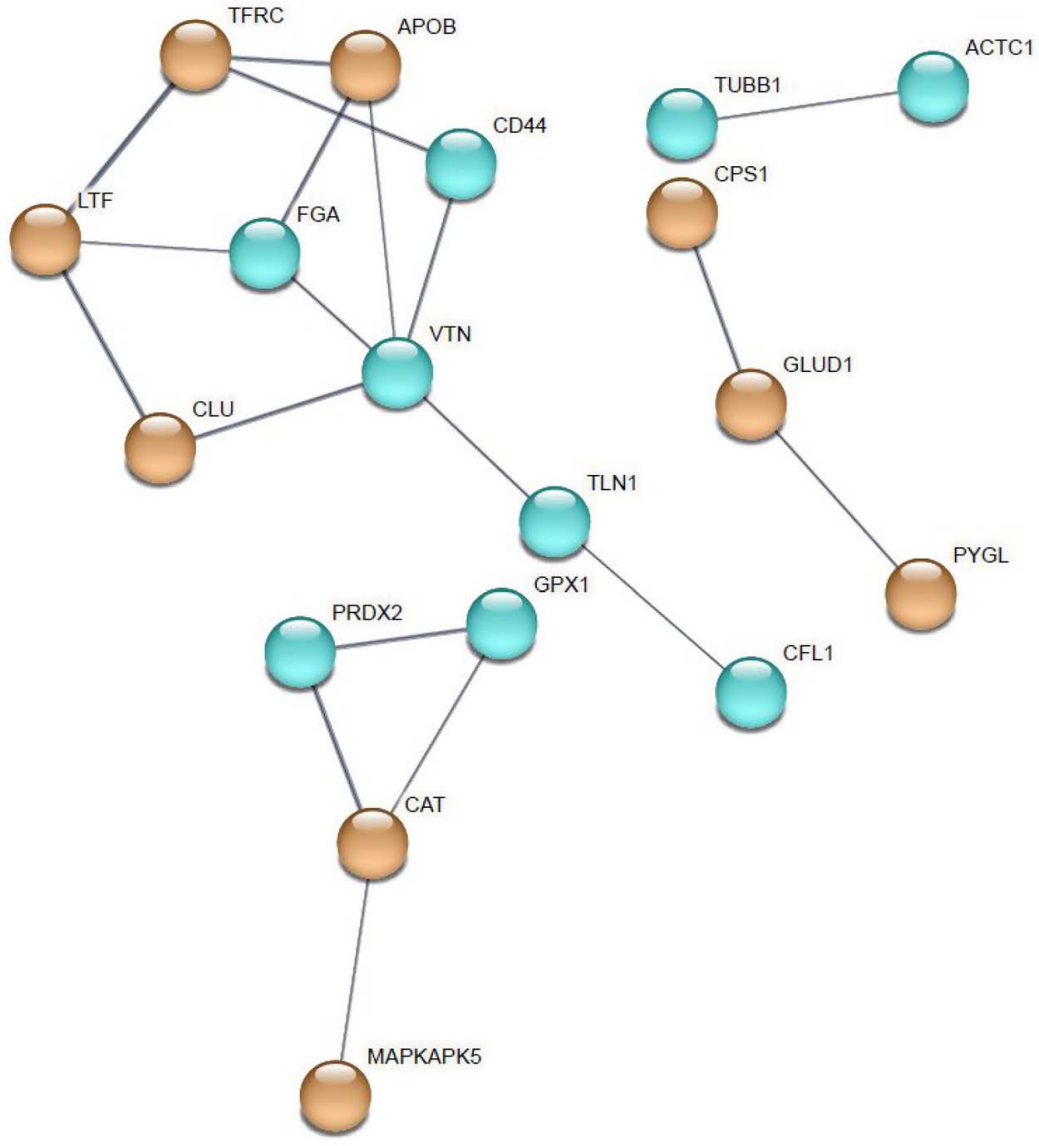
Protein–protein interaction network for differentially abundant plasma proteins in dogs with pheochromocytomas (PCC) vs healthy controls. Nodes represent proteins (blue: higher levels in dogs with PCC; orange: lower levels in dogs with PCC), and edges indicate interaction confidence, where line thickness reflects the strength of data support. Disconnected nodes are hidden.

STRING analysis revealed an interconnected network of differentially abundant proteins with small clusters spanning adhesion, hemostasis, redox/stress, and metabolism (Figure 5).

### Enrichment analysis

**Figure 6:**
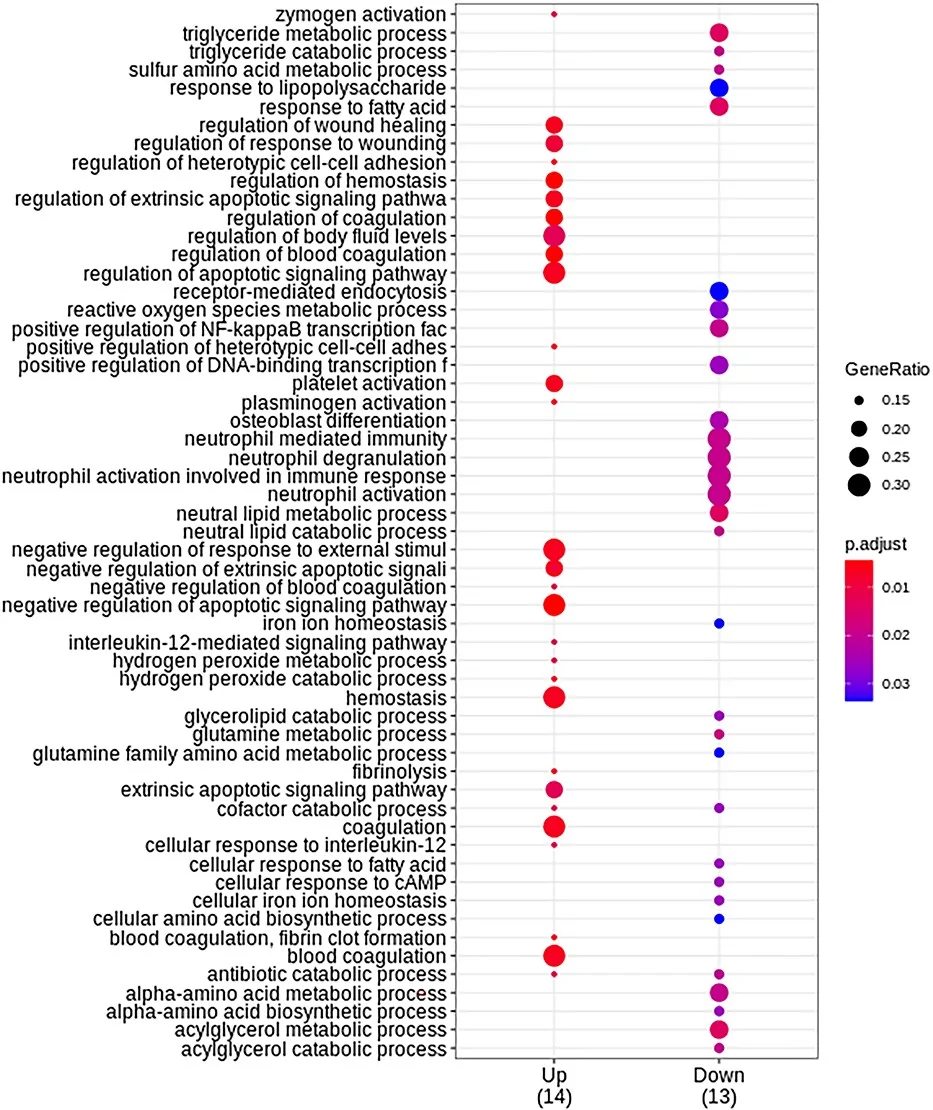
Enrichment analysis, showing comparison of enriched biological processes among proteins with increased and decreased abundance. Each dot represents an enriched GO term; its color indicates the adjusted *P* value (Benjamini–Hochberg correction, from blue for less significant to red for most significant), and its size reflects the number of differentially abundant proteins associated with that term. The gene ratio represents the proportion of significant proteins relative to the total number of proteins annotated to the respective GO term.

**Figure 7:**
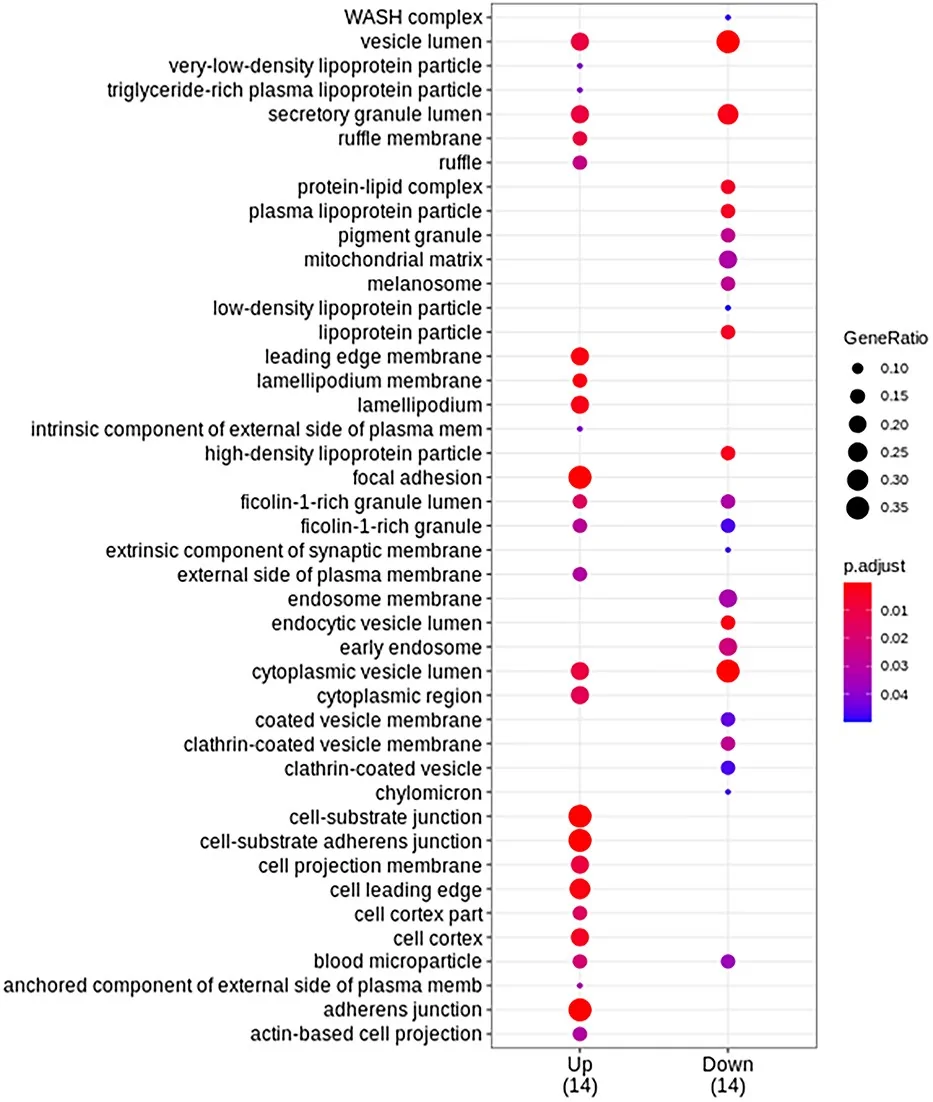
Enrichment analysis, showing comparison of enriched cellular components among proteins with increased and decreased abundance. Each dot represents an enriched gene ontology (GO) term; its color indicates the adjusted *P* value (Benjamini–Hochberg correction, from blue for less significant to red for most significant), and its size reflects the number of differentially abundant proteins associated with that term. The gene ratio represents the proportion of significant proteins relative to the total number of proteins annotated to the respective GO term.

In both SEA and MEA, no GO terms reached statistical significance after adjustment for multiple testing in any of the 3 domains (BP, MF, CC). In contrast, direction-specific analysis using the Biological theme comparison (clusterProfiler) of subsets of proteins with increased and decreased abundance revealed significant functional differences in plasma proteomes between dogs with PCC and healthy controls. Proteins with increased abundance were predominantly enriched in biological processes related to hemostasis and regulation of apoptotic signaling, whereas those with lower abundance showed enrichment in lipid and amino acid metabolism, oxidative stress response, and neutrophil activation (Figure 6). In the CC domain, proteins showing elevated abundance were significantly enriched in structures related to cell adhesion and cytoskeletal organization, including focal adhesion, adherens junctions, and lamellipodium membranes, consistent with enhanced cell–matrix and cell–cell interaction potential. In contrast, proteins with decreased abundance were mainly associated with vesicle and lipoprotein particles (Figure 7). These enrichment results provide functional context for the observed protein changes and should be interpreted as hypothesis-generating rather than definitive pathway-level evidence, requiring validation before firm conclusions can be drawn.

## Discussion

This study characterizes the plasma proteomic profile of dogs with PCC. Quantitative proteomic profiling revealed separation between PCC-affected and healthy control dogs, indicating distinct protein abundance profiles. In total, 261 proteins were reliably quantified, of which 51 showed differential abundance based on an FDR-adjusted *P* < .05. Among these, 15 proteins were increased, and 18 were decreased in abundance (log₂ FC > 1 or < −1). Compared with healthy controls, dogs with PCC showed elevated plasma levels of proteins implicated in tumor progression, cell adhesion, and migration-related pathways, as well as alterations in oxidative stress and metabolic processes.

Given that the PCC and control groups were not matched for age, sex, breed, or comorbidities, and that the PCC cohort was clinically heterogeneous, these findings should be interpreted as exploratory and hypothesis-generating. As a result, some of the observed differences in protein abundance might reflect these factors rather than PCC-specific biological processes and require validation in larger, well-matched cohorts.

Several of the proteins with increased plasma abundance in dogs with PCC have previously been implicated in tumor progression, particularly through their roles in cell motility and metastatic behavior. Cofilin 1, an actin-binding protein, is frequently overexpressed in human cancers and has been associated with invasion, metastasis, and poor prognosis.^18–20^ It promotes cell motility by regulating cytoskeletal reorganization, enhancing lamellipodium formation, and facilitating epithelial-to-mesenchymal transition (EMT).^19^^,^^20^ CD44, a cell-surface glycoprotein involved in cell–cell interactions, adhesion, and migration, is recognized as a marker for cancer stem cells and plays a central role in tumor progression by mediating interactions with the extracellular matrix, promoting EMT, and facilitating invasion and metastasis.^21^^,^^22^ Talin 1, a cytoskeletal protein linking integrins to the actin cytoskeleton, is highly expressed in various cancers and contributes to tumor cell invasiveness, proliferation, and metastatic progression.^23^ Lastly, cathepsin Z, a cysteine protease, promotes tumor cell motility and metastasis by modulating EMT markers and upregulating matrix-degrading proteases, and might also act via non-proteolytic mechanisms.^24^^,^^25^ Together, these proteins reflect a coordinated activation of migration-related pathways in PCC and might serve as indicators of metastatic behavior. Several of these proteins, including cofilin-1, CD44, and talin 1, have also been proposed as therapeutic targets in other tumor types, supporting their potential relevance for future studies in PCC.^20–23^

Although metastatic disease was not documented at diagnosis in our PCC cohort, 1 dog (PCC9) developed extensive local recurrence with vascular invasion and multifocal renal and nodal metastases approximately 2.5 years after adrenalectomy, culminating in euthanasia. Vascular invasion was present at diagnosis in 3 dogs; however, in this small cohort, plasma levels of migration/adhesion-related proteins (including CD44, cofilin-1, and talin 1) did not show a consistent association with vascular invasion status. Given this single metastatic event and the limited sample size, together with the absence of longitudinal plasma profiling, no prognostic inferences can be drawn. Prospective studies with serial sampling are needed to determine whether baseline levels or temporal trajectories of migration/adhesion-related proteins associate with vascular invasion, recurrence, or metastatic progression. Complementary validation in tumor tissue could help clarify whether circulating changes reflect tumor-intrinsic expression or systemic responses.

Among proteins with decreased plasma abundance, clusterin showed the largest reduction. Although clusterin is often elevated in cancer,^26^^,^^27^ reduced clusterin has also been reported and linked to poor prognosis,^28^ and lower serum clusterin has been described in canine lymphoma, underscoring context-dependent regulation.^29^ Of interest, reduced clusterin secretion has been reported in von Hippel–Lindau (*VHL*)-mutant PCC models via a hypoxia-inducible factor-independent mechanism.^30^ In human PCC, alterations in the *VHL* pathway are well established,^31^ whereas *VHL* status has not been assessed in dogs with PCC and the genetic landscape of canine PCC remains incompletely defined. Although tumor genotyping was not available for the present cohort, dysregulation of *VHL*-related pathways could contribute to the lower circulating clusterin observed here and warrant further investigation in future canine PCC studies.

In parallel, glutamate dehydrogenase 1, which converts glutamate into α-ketoglutarate, a key intermediate of the tricarboxylic acid (TCA) cycle, showed markedly lower plasma abundance. The TCA cycle is known to play a central role in the pathogenesis of human PCCs, particularly in tumors with mutations in succinate dehydrogenase subunits (*SDHx*).^31^^,^^32^ In dogs, a previous study reported lower intratumoral glutamate in PCC compared with normal adrenal tissue.^33^ Taken together, reduced circulating glutamate dehydrogenase 1and tissue glutamate depletion point to perturbations of the glutamine–glutamate–TCA axis. Targeted metabolomics and paired tumor–plasma analyses could provide complementary insight into the relationship between circulating and intratumoral metabolic alterations in the glutamine–glutamate–TCA axis.

In addition to the migration-associated proteins, 2 plasma proteins with increased abundance were linked to oxidative stress response**:** peroxiredoxin 2 and glutathione peroxidase 1. Oxidative stress is a hallmark of cancer, and tumor cells often adapt to elevated reactive oxygen species (ROS) by upregulating antioxidant defense mechanisms, which can contribute to therapy resistance.^34^ Peroxiredoxin 2 is overexpressed in various human solid tumors and plays a broader role in cancer biology, promoting tumor cell survival, invasion, and resistance to apoptosis.^34^ Similarly, glutathione peroxidase 1 is a key antioxidant enzyme that is abnormally expressed in many cancer types, although its role appears to be dependent on the tumor context. While protein-level expression of peroxiredoxin 2 and glutathione peroxidase 1 has not been specifically studied in PCC tissue, available transcriptomic data from both human (http://gepia2.cancer-pku.cn) and canine tumors do not indicate increased expression in tumor cells.^35^ Their elevated plasma abundance might therefore reflect a systemic antioxidant response to tumor-induced oxidative stress, rather than tumor-intrinsic overexpression. The observation that catalase, another major antioxidant enzyme, showed reduced plasma abundance in dogs with PCC might seem paradoxical. However, decreased catalase expression has been reported in various human cancers and is often associated with increased proliferation and angiogenesis.^36^ While catalase protects cells from oxidative damage by degrading hydrogen peroxide, reduced activity might enable tumor cells to maintain sublethal ROS levels that promote growth and signaling.^36^ Viewed as a whole, the differential plasma levels of these antioxidant enzymes, with some being increased and others decreased, underscore the complex and context-dependent nature of redox regulation in cancer.

From a clinical perspective, redox-regulating enzymes such as peroxiredoxin 2and glutathione peroxidase 1represent potential therapeutic targets. Several preclinical studies in human oncology have demonstrated that inhibition or knockdown of peroxiredoxin 2 increases intracellular ROS, inhibits tumor cell growth, and sensitizes cancer cells to chemotherapy and radiotherapy.^34^ Recently, the covalent peroxiredoxin 2 inhibitor Conoidin A was shown to reduce glioblastoma cell viability through ROS accumulation.^37^ Similar strategies targeting the glutathione system, including glutathione peroxidase 1inhibitors, are also being explored as anticancer approaches.^38^ Although such interventions have not yet been investigated in PCC, the upregulation of circulating antioxidant enzymes observed here suggests that targeting redox homeostasis could represent a promising therapeutic avenue.^39^

Both peroxiredoxin 2 and peptidylprolyl isomerase A are identified as tumor-associated antigens capable of eliciting serum autoantibody responses in patients with early-stage breast cancer.^40^^,^^41^ Autoantibody panels including these proteins have shown promise in distinguishing early breast cancer from healthy controls.^40^^,^^41^ The elevated plasma concentrations of peroxiredoxin 2 and peptidylprolyl isomerase A observed in dogs with PCC might similarly reflect their release as circulating tumor-associated antigens. Although the present findings demonstrate increased antigen rather than antibody levels, they highlight the potential diagnostic relevance of these proteins. Further studies should evaluate whether peroxiredoxin 2 and peptidylprolyl isomerase A, either as antigen biomarkers (alone or within a broader panel) or in combination with their corresponding autoantibodies, could aid in early detection, postoperative monitoring, or serve as targets for immunomodulatory interventions in canine PCC.

In addition to peroxiredoxin 2 and peptidylprolyl isomerase A, CD44 stands out given its relevance in tumor biology and its dual potential as a circulating biomarker and therapeutic target. However, CD44 is not unique to PCC; increased expression has been reported in various canine tumor types, including mammary tumors.^42^ Moreover, elevated serum CD44 concentrations have been described in dogs with lymphoma, and increased CD44 mRNA and protein expression have been demonstrated in dogs with acute and chronic leukemia.^43^^,^^44^ This study reports elevated plasma CD44 concentrations in dogs with a solid tumor, warranting further investigation into its potential as a circulating biomarker. Nevertheless, as CD44 upregulation has been observed in different neoplastic contexts, elevated plasma levels might not be specific for PCC and might instead reflect a general cancer-associated signal. Beyond this, CD44 also represents an interesting therapeutic target. In human oncology, the CD44 variant isoform v6 has been targeted with the monoclonal antibody bivatuzumab, which showed selective binding to tumor cells in preclinical studies, although clinical development was discontinued due to toxicity.^45^ In dogs with PCC, RNA sequencing did not indicate increased *CD44* transcript levels.^35^ Further evaluation of CD44 protein expression in tumor tissue is warranted.

In the context of human PCC proteomics, where blood-based studies are scarce,^46^ 1 earlier serum study using SELDI-TOF reported low-molecular-weight spectral patterns that discriminated metastatic from benign disease in a blinded set but did not identify the underlying proteins.^47^ In contrast, our LC–MS approach identifies circulating proteins within a pathway context. Other human PCC proteomic investigations have been tissue-based rather than blood-based,^48^^,^^49^ limiting direct comparison to our circulating proteome readout.

This study has several limitations. First, the sample size was small, limiting statistical power and generalizability, as is common in exploratory proteomics studies. Second, longitudinal sampling was not performed, precluding assessment of temporal changes in protein abundance in relation to disease stage, postoperative course, or recurrence. Third, only plasma was analyzed; matched tumor and normal adrenal tissues and tumor genotyping (eg, *SDHx*/*VHL* mutations) were not available, limiting tissue–plasma correlation. Fourth, functional enrichment analyses were exploratory and annotation-dependent; therefore, causal relationships between altered proteins and PCC pathogenesis cannot be inferred. Fifth, groups were not matched for sex, breed, or age (with PCC dogs being older), introducing potential confounding. Age might have influenced the plasma proteomic profile observed in this study, as age-related changes in circulating proteins have been described in dogs, including alterations in routine biochemical protein parameters (eg, total protein, albumin, and globulins)^50^ and, more recently, in plasma proteomic profiles.^51^ However, these studies did not report age-associated changes in the specific proteins identified in the present study. Nevertheless, subtle age-related effects on their plasma abundance cannot be excluded and should be considered when interpreting the results. In addition, several PCC-affected dogs had comorbidities at the time of sampling, which might have influenced circulating protein profiles. Sixth, baseline clinicopathologic data (eg, CBC/biochemistry) were not collected in a standardized manner across sites and were incomplete, precluding meaningful comparison between PCC and control groups. Finally, the PCC cohort was clinically heterogeneous, including both biochemically active and biochemically negative tumors. While this might have increased variability, it was an intentional choice to enhance clinical relevance and to facilitate discovery of circulating, catecholamine-independent biomarkers that could aid diagnosis and therapeutic targeting of biochemically negative PCCs.

In conclusion, this work characterizes the plasma proteomic profile of dogs with PCC and shows a distinct circulating protein signature relative to healthy controls. We identified 51 differentially abundant proteins. Proteins with increased abundance were involved in cell adhesion and migration (cofilin-1, CD44, talin 1, peptidylprolyl isomerase A, cathepsin Z), apoptotic regulation (peroxiredoxin 2, fibrinogen α-chain, CD44, glutathione peroxidase 1), and redox regulation (peroxiredoxin 2, glutathione peroxidase 1). Proteins with reduced abundance were centered on iron handling (transferrin receptor 1, lactotransferrin), lipoprotein transport (apolipoprotein B), antioxidant defense and detoxification (catalase, aldehyde dehydrogenase 2), and enzymes of the glutamine–glutamate and nitrogen-metabolism axis (glutamate dehydrogenase 1, adenosylhomocysteinase, carbamoyl-phosphate synthase 1). Collectively, this proteomic profile provides clues to PCC pathogenesis, pointing to enhanced cell–matrix interactions and perturbed redox and metabolic homeostasis, and nominates several circulating proteins (eg, CD44, peroxiredoxin 2, glutathione peroxidase 1, peptidylprolyl isomerase A) as candidates for diagnostic panels and as starting points for therapeutic exploration.

## Supplementary Material

Supplementary_material_aalag212

## Data Availability

The data underlying this article will be shared at reasonable request to the corresponding author.

## Off-label antimicrobial declaration

Authors declare no off-label use of antimicrobials.

## Appendix

### Materials and methods

#### Study population and sample collection

This multicenter, retrospective observational study compared plasma proteomic profiles between dogs with PCC and healthy controls. Plasma from 10 client-owned dogs with PCC was included. Samples were obtained as surplus clinical material obtained during routine diagnostic or pre-anesthetic blood sampling at Utrecht University’s Department of Clinical Sciences, Faculty of Veterinary Medicine (*n* = 4), University of Bologna (*n* = 3), and veterinary referral centers in the Netherlands (*n* = 3). Written informed consent was obtained from all owners before participation. PCC diagnosis was confirmed by histopathology and IHC using adrenomedullary markers chromogranin A (CHGA) and synaptophysin (SYP).^35^ Medical records were retrospectively reviewed and clinicopathological features recorded for each dog included breed, age, sex/neutering status, clinical signs, comorbidities, biochemical findings (eg, plasma/urinary metanephrines), tumor laterality and size, presence of vascular invasion or tumor thrombus, presence of metastasis, treatment (surgery/medical), histopathology and IHC (CHGA, SYP, Ki67), and follow-up/survival when available.

For the control group, surplus plasma from 10 clinically healthy, client-owned dogs with no signs of illness in the past 2 months was used. These dogs were presented for clinical examination and blood sampling at the Clinic for Small Animal Internal Medicine (Veterinary University of Vienna, Austria) over a 6-month period. They were also enrolled in a behavioral study (Ref: BMBWF 20221-0.210.26) conducted by the Interuniversity Messerli Research Institute at the same university. Written informed consent was obtained from all owners before participation. Dogs of various breeds, body weights, and genders, aged 1-10 years, were included. Eligibility was determined through a comprehensive assessment, including a detailed medical history, physical examination, and blood sampling, including CBC and serum or plasma biochemical profiling. Exclusion criteria were age <1 year, body weight <3 kg, any clinical abnormalities, or medication use within the past 2 months. Dogs with significant deviations in any blood parameters were also excluded. All procedures followed relevant Austrian guidelines and regulations, and the study adhered to the ARRIVE guidelines (https://arriveguidelines.org).

#### Quantitative proteome analysis by label-free LC–MS

##### Sample preparation

Plasma was stored at -20 °C. For protein extraction, 5 μL of plasma was transferred to a tube containing 20 μL of urea denaturing buffer (6 M urea, 2 M thiourea, 10 mM HEPES; pH 8.0). Disulfide bonds were reduced with 1 μL of 10 mM dithiothreitol and incubated at room temperature for 30 minutes. Alkylation was performed by adding 1 μL of 55 mM iodoacetamide, followed by another 30-min incubation in the dark. Samples were then diluted with 4 volumes of 40 mM ammonium bicarbonate buffer and digested overnight at 37 °C with 1 μL of trypsin protease (1 μg/μL, Thermo Scientific, United States). Afterwards, 5% acetonitrile and 0.3% trifluoroacetic acid (TFA) were added to acidify the digested samples. Peptides were then desalted using C18 StageTips with Empore™ C18 Extraction Disks. Before MS analysis, the eluted peptides were dried via vacuum centrifugation and reconstituted in 20 μL of 0.05% TFA and 4% acetonitrile.

##### Mass spectrometry analysis

One microliter of each sample was injected into an Ultimate 3000 nano-LC system coupled to a Q Exactive HF mass spectrometer (Thermo Fisher Scientific). Samples were concentrated on a PepMap100 C18 trap column (3 μm, 100 Å, 75 μm i.d. × 20 mm, Thermo Scientific) equilibrated with 0.05% TFA. After switching the trap column inline, peptides were separated using an Acclaim PepMap100 C18 capillary column (2 μm, 100 Å, 75 μm i.d. × 250 mm, Thermo Scientific) at a flow rate of 300 nL/min. Mobile phase A consisted of 0.1% formic acid in water, while mobile phase B contained 0.1% formic acid and 80% acetonitrile in water. The column was pre-equilibrated with 5% mobile phase B, followed by a gradient increase to 44% B over 100 minutes. Data acquisition was performed in data-dependent mode with a full MS scan (m/z 350-1650) at a resolution of 60,000, followed by MS/MS scans of the 15 most intense precursor ions at a resolution of 15,000. Dynamic exclusion was set to 20 seconds, and the automatic gain control target values were 3 × 10^6^ for MS scans and 1 × 10^5^ for MS/MS scans.

#### Proteomics data processing and statistical analysis

Raw MS data were analyzed using MaxQuant (version 2.6.6.0) with the integrated Andromeda search engine.^12^ Data were searched against the *C. lupus familiaris* reference proteome (ID: UP000805418; downloaded from UniProt on September 20, 2024, containing 43,623 sequences). Default settings were used with label-free quantification (LFQ) and the match-between-runs feature enabled, applying default FDR thresholds of 1% at both peptide and protein levels. Data filtering and statistical analysis were performed using Perseus (version 2.1.3.0). Proteins with LFQ intensity values present in at least 3 of 10 replicates in at least 1 experimental group were included in the analysis, providing an additional quality filter. Missing values were imputed from a normal distribution (width: 0.3, downshift: 1.8). Standardized sample preparation, LC–MS acquisition, and data filtering procedures were used throughout the workflow to ensure analytical quality.

Differentially abundant proteins were identified using Student’s *t-*test in Perseus.^13^ Proteins with a log₂ FC >1 or < −1 and an FDR-adjusted *P*-value ≤ .05 were considered significantly abundant. All statistical analyses were performed using R (version 4.4.2). Normality was assessed via the Shapiro–Wilk test. Non-normally distributed variables were analyzed using the Mann–Whitney U test, while normally distributed data were compared using analysis of variance (ANOVA).

#### Clinical data statistical analysis

Data analysis was performed by the statistical software program IBM SPSS Statistics for Windows, version 29 (IBM Corp., Armonk, NY, United States). Data were assessed for normality of distribution by the Shapiro–Wilk test. Differences in results between 2 groups were assessed by the independent samples *t-*test or Mann–Whitney *U*-test. Values were considered significant at *P* < .05.

#### Bioinformatics

##### Gene ontology classification

Gene ontology classification was performed using PANTHER to investigate the functional significance of proteins showing differential abundance in dogs with PCC compared to the healthy control group.^14^ Only proteins that showed significantly increased or decreased abundance in the PCC group (with an FDR-adjusted *P* < .05 and log_2_ FC of >1 or < −1) were included. The analysis included categorization by BP, MF, protein class, and pathway.

##### Protein interaction network

Protein–protein interaction analysis was performed using the STRING database (version 12.0; https://string-db.org)^15^ for *C. lupus familiaris*. Proteins with differential abundance were uploaded to construct interaction networks based on STRING connectivity algorithms, with a medium confidence score (0.4).

##### Enrichment analysis

To statistically assess functional enrichment, GO term enrichment analysis was performed using the Proteomics Research Environment (ProteoRE), a Galaxy-based platform.^16^^,^^17^ Proteins showing increased or decreased abundance (log_2_ FC of >1 or < −1, FDR-adjusted *P* < .05) were compared to a reference set of proteins comprising all identified and quantified proteins. Both SEA and MEA were applied for the GO domains BP, MF, and CC, with multiple testing correction using the Benjamini–Hochberg method. To explore direction-specific functional differences, protein subsets with increased and decreased abundance were compared through GO enrichment analyses of BP, MF, and CC using the Biological theme comparison (clusterProfiler) tool in ProteoRE.

## Abbreviations

BP: biological process
CC: cellular component
CHGA: chromogranin A
EMT: epithelial-to-mesenchymal transition
FC: fold change
FDR: false discovery rate
GO: gene ontology
IHC: immunohistochemistry
LC–MS: liquid chromatography-mass spectrometry
MEA: Modular Enrichment Analysis
MF: molecular function
PCC: pheochromocytoma
ROS: reactive oxygen species
SDHx: succinate dehydrogenase subunits
SEA: Singular Enrichment Analysis
SYP: synaptophysin
TCA: tricarboxylic acid
TFA: trifluoroacetic acid
VHL: von Hippel–Lindau
