# Supplementary material for: Plasma proteomic profiling in dogs with pheochromocytoma
Source: J Vet Intern Med. 2026 Sep 21;40(5):aalag212. doi: 10.1093/jvimsj/aalag212 (PMC13592768; doi:10.1093/jvimsj/aalag212)
Supplement: Supplementary file 1 [file supplementary_material_aalag212.zip › Supplementary_material_aalag212/Supplementary Figure 1.docx]

**Supplementary Figure 1**. Heatmap of plasma proteins showing higher levels in dogs with PCC relative to healthy controls. A total of 14 proteins, with a log₂ fold-change of >1, are represented. Blue indicates lower relative abundance, and red indicates higher abundance.
