# Supplementary material for: Plasma proteomic profiling in dogs with pheochromocytoma
Source: J Vet Intern Med. 2026 Sep 21;40(5):aalag212. doi: 10.1093/jvimsj/aalag212 (PMC13592768; doi:10.1093/jvimsj/aalag212)
Supplement: Supplementary file 1 [file supplementary_material_aalag212.zip › Supplementary_material_aalag212/Supplementary Table 1.docx]

**Supplementary Table 1.** Signalment and clinicopathologic features of dogs with PCC included in the proteomic analysis.

| Patient number | Breed | Sex | Age (y) | pNMN (nmol/L) | pMN (nmol/L) | Tumor side and size | Vascular invasion |
| --- | --- | --- | --- | --- | --- | --- | --- |
| PCC1 | Shih Tzu | MN | 11.7 | NA | NA | Left, 2.1 cm | No |
| PCC2 | Miniature Pinscher | FN | 13.0 | 6.4 | 2.9 | Left, 2.3 cm | No |
| PCC3 | Cane Corso | ME | 11.4 | >54 | 10 | Left, 7.1 cm | No |
| PCC4 | Australian Labradoodle | MN | 10.9 | 21 | 4.0 | Left, 2.2 cm | Yes, v. phrenicoabdominalis |
| PCC5 | English Cocker Spaniel | FN | 9.6 | 3.55 | 0.52 | Right, 3.3 cm | No |
| PCC6 | Cavalier King Charles Spaniel | ME | 11.8 | NA | NA | Right, 6.2 cm | Yes, caudal vena cava |
| PCC7 | Labrador Retriever | ME | 11.5 | 10.5 | 1.0 | Left, 1.0 cm | No |
| PCC8 | Staffordshire Bull Terrier | FN | 10.0 | 6.1 | 5.3 | Left, 4.0 cm | No |
| PCC9 | Border Terrier | ME | 10.0 | 3.7 | 2.8 | Left, 1.8 cm | No |
| PCC10 | Norfolk Terrier | FN | NA | NA | NA | Right, 3.0 cm | Yes |

PCC, pheochromocytoma; y, years; pNMN, plasma normetanephrine; pMN, plasma metanephrine; NA, not available.

Reference intervals: pNMN: 0.90-3.56 nmol/L; pMN: 0.35-2.49 nmol/L.^1^

Reference

1. van den Berg MF, Kooistra HS, Grinwis GCM, van Nimwegen SA, van Faassen M, Kema IP, et al. Reference intervals for plasma, urinary, and salivary concentrations of free metanephrines in dogs: Relevance to the diagnosis of pheochromocytoma. *J Vet Intern Med*. 2023;37(1):173-183.
